# Contact Lenses for Keratoconus- Current Practice

**DOI:** 10.2174/1874364101711010241

**Published:** 2017-07-31

**Authors:** Marilita M. Moschos, Eirini Nitoda, Panagiotis Georgoudis, Miltos Balidis, Eleftherios Karageorgiadis, Nikos Kozeis

**Affiliations:** 1 Department of Ophthalmology, Medical School, National & Kapodistrian University of Athens, Greece; 2 Queen Victoria Hospital NHS Trust, East Grinstead, United Kingdom.; 3 Institute of Ophthalmology and Ocular Microsurgery ‘Ophthalmica’, Thessaloniki, Greece

**Keywords:** Keratoconus, Cornea, Contact lens, Visual rehabilitation, RMS, GP

## Abstract

**Background:**

Keratoconus is a chronic, bilateral, usuallly asymmetrical, non-inflammatory, ectatic disorder, being characterized by progressive steepening, thinning and apical scarring of the cornea. Initially, the patient is asymptomatic, but the visual acuity gradually decreases, resulting in significant vision loss due to the development of irregular astigmatism, myopia, corneal thinning and scarring. The classic treatment of visual rehabilitation in keratoconus is based on spectacles and contact lenses (CLs).

**Objective:**

To summarize the types of CLs used in the treatment of keratoconus. This is literature review of several important published articles focusing on the visual rehabilitation in keratoconus with CLs.

**Method:**

Gas permeable (GP) CLs have been found to achieve better best corrected visual acuity than spectacles, eliminating 3rd-order coma root-mean-square (RMS) error, 3rd-order RMS, and higher-order RMS. However, they have implicated in reduction of corneal basal epithelial cell and anterior stromal keratocyte densities. Soft CLs seem to provide greater comfort and lower cost, but the low oxygen permeability (if the lens is not a silicone hydrogel), and the inability to mask moderate to severe irregular astigmatism are the main disadvantages of them. On the other hand, scleral CLs ensure stable platforms, which eliminate high-order aberrations and provide good centration and visual acuity. Their main disadvantages include the difficulties in application and removal of these lenses along with corneal flattening and swelling.

**Result:**

The modern hybrid CLs are indicated in cases of poor centration, poor stability or intolerance with GP lenses. Finally, piggyback CL systems effectively ameliorate visual acuity, but they have been related to corneal neovascularization and giant papillary conjunctivitis.

**Conclusion:**

CLs seem to rehabilitate visual performance, diminishing the power of the cylinder and the high-order aberrations. The final choice of CLs is based on their special features, the subsequent corneal changes and the patient’s needs.

## 1 INTRODUCTION

Keratoconus (KCN) is a chronic, bilateral, usuallly asymmetrical, non-inflammatory, ectatic disorder, being characterized by progressive steepening, thinning and apical scarring of the cornea [1]. Its prevalence was considered to be 1 in every 2000 individuals until the development of corneal topography devices, which raised the number to 54, 1190, 2300, 3.3 and 20 per 100,000 in the USA, France, India, Iran (Tehran) and Middle East, respectively [1-3]. Structural abnormalities in the corneal epithelium, Bowman’s layer and mainly stromal collagen along with altered tear components are responsible for its clinical features. The latter include declined focusing at all distances due to progressive myopia and irregular astigmatism, increased corneal high order aberrations (mainly coma) affecting primarily visual quality, inducing haloes and image distortions. These visual disorders may have major impact on the quality of life [1, 2]. Keratometric, retinoscopic or slit lamp findings are important diagnostic tools for clinical keratoconus, while the subclinical type exhibits only mild topographic changes [1].

### 1.1 Pathogenesis of Keratoconus

Biomechanics, enzymology, proteomics, and molecular genetics are implicated in the pathogenesis of keratoconus. Altered expression of extracellular matrix proteoglycans, such as decorin, lumican, biglycan and keratocan, and proteins, along with decreased stromal collagen content are the basic structural changes observed in keratoconus [4, 5]. The changes in extracellular components result in the distortion of collagen fibers and lamellae, eliminating corneal strength and transparency [4, 5]. Additionally, alteration in cell junctions are associated with decreased levels of transforming growth factor beta (TGF-β) [5]. Accumulation of proteolytic enzymes, including cathepsin-B, -G, -V/L2 and lysosomal enzymes is implicated in degradation of collagen and cell death [5]. Furthermore, cathepsins regulate cell apoptosis and mitochondria function, contributing to oxidative stress. Increased mtDNA content in patients with keratoconus may indicate mitochondrial respiratory chain defects [6]. An imbalance between matrix metalloproteinases-2 (MMPs-2) and their tissue inhibitors (TIMPs), which exhibits anti-apoptotic properties, has also been related to corneal thinning. The expression of MMPs and cell apoptosis are impaired by the high levels of interleukins, which are released by eye rubbing and chronic contact lenses wearing. High levels of transforming growth factor (TGF-β1), and dual-specificity phosphates 1 (DUSP1) messenger ribonucleic acid (mRNA) have also been measured in eyes with keratoconus [7].

Although keratoconus is usually sporadic, a genetic predisposition with increased incidence in familial and monozygotic twins has been described [4, 8]. The modes of keratoconus inheritance are dominant and recessive, but in autosomal dominant inheritance, the disease shows incomplete penetrance with variable phenotype [8]. Moreover, it has been related to systemic and ocular conditions, including Leber congenital amaurosis, anterior polar cataract, Down syndrome (10–300-fold higher prevalence) and Ehlers Danlos syndrome [4]. VSX1 (Visual system homeobox 1), TIMP3, TGFBI, ZEB1, filaggrin (*FLG*) and several collagen genes, such as COL4A3, COL4A4 AND COL5A1, have been associated with keratoconus, while the distinct potential loci, which have been described, include 3p14-q13, 5q21, 5q32 and 14q11. Furthermore, LOX, FOXO1, FNDC3B, RXRA-COL5A1, MPDZ-NF1B, COL5A1 and ZNF469 are single-nucleotide polymorphisms (SNPs), exhibiting high risk of keratoconus [4, 5, 8].

Environmental factors, including eye rubbing, atopy, and sun (UV) exposure have been implicated in pathogenesis of keratoconus [9]. Moreover, a positive familial history and the parental education and socioeconomic status seem to impair the development of the disease. Keratoconus exhibits different distribution, depending on geographic location and race. Asians (Indians, Bangladeshi, and Pakistani) living in the English Midlands have an incidence of the disease 4.4 times higher than in whites. Moreover, Northern Europe, Japan and the Urals have low prevalence, as well as northern USA. On the other hand, it is relatively high in the countries of Middle East, India and China. Finally, the age seems to affect the development of keratoconus; the mean diagnostic age ranges from 20.0 years (±6.4) to 24.05, whereas it seldom appears after the age of 45 years [9].

### 1.2 Clinical Features and Diagnosis

Keratoconus is a progressive condition which usually stabilizes by the fourth to fifth decade of life [9]. Initially, the patient is asymptomatic, but the visual acuity gradually decreases, resulting in significant vision loss due to the development of irregular astigmatism, myopia, corneal thinning and scarring. Although the disease develops unilateral, the majority of patients eventually develop bilateral keratoconus [9]. The early *signs* of keratoconus include Fleischer's ring, which is a partial or complete circle of iron deposition in the epithelium surrounding the base of the cone and Vogt's striae, fine vertical lines produced by compression of Descemet's membrane. Oil droplet reflex can be seen with direct ophthalmoscope, while retinoscopy reveals an irregular scissor reflex. As the disease progresses, a Munson's sign appears, being characterized by a V-shaped deformation of the lower lid, when the patient looks downwards [10]. Rizzuti's sign is another feature of disease progression and it represents a bright reflection of the nasal area of the limbus [11]. Less common are breaks in Descemet's membrane, resulting in hydrops, which are described by stromal edema, vision loss, and associated pain [10].Corneal scarring is a common sign of wearing contact lenses [12].

The basic diagnostic examinations for keratoconus include placido disk–based corneal topography, Orbscan I (Bausch & Lomb, Rochester, New York, USA) and II slit topography, Pentacam (Oculus, Wetzlar, Germany) Scheimpflug imaging, wavefront aberrometers and spectral domain optical coherence tomography (SD-OCT). On the other hand, confocal microscopy, Ocular Response Analyzer (ORA, Reichert Inc., Depew, New York, USA) and Fourier Transform Infrared (FTIR) spectroscopy are more advanced diagnostic devices, not specific for keratoconus and still under investigation. The Placido videokeratography map of keratoconus displays a zone of increased corneal power surrounded by zones of decreasing corneal power, inferior-superior asymmetry in corneal power and skewing of the steepest radial axes above and below the horizontal meridian [13]. Although this is a highly sensitive and specific diagnostic tool, it only evaluates the anterior surface of the cornea.

The anterior and posterior corneal elevation as measured by Orbscan II could be useful in differentiating clinical and possible subclinical keratoconus from normal eyes [14]. Jafarinasab et al. estimated the mean posterior corneal elevation to be 106.80 ± 43.98 μm, 36.60 ± 22.80 μm and 25.00 ± 9.15 μm in clinical and subclinical keratoconus and in normal eyes, respectively. 49.35 ± 21.60 μm, 15.07 ± 7.48 μm and 11.05 ± 4.03 μm were the values of mean anterior corneal elevation μm in clinical and subclinical keratoconus and in normal eyes, respectively [14]. A cutoff point of ≥ 51 μm was highly sensitive (89.23%) and specific (98.58%) for posterior corneal elevation and a cutoff point of ≥ 19 μm was highly sensitive (93.85%) and specific (97.16%) for anterior corneal elevation to differentiate clinical keratoconus from normal subjects [14].

The Scheimpflug imaging used by Pentacam provides a combination of corneal refractive (keratometric), topometric, tomographic, and pachymetric data. It can evaluate the indices of surface variance (measure of corneal surface irregularity), vertical asymmetry (value of curvature symmetry), height asymmetry (the difference between superior and inferior height values), height decent ration (the value of the decent ration of elevation data in the vertical direction), minimum radius of curvature (measurement of the smallest radius of sagittal corneal curvature), keratoconus index (the ratio between mean radius values in the upper and lower segment) and central keratoconus index, as well [15]. Kanellopoulos et Asimellis supported that these indices of surface variance and of height asymmetry are more sensitive and specific tools to see visual acuity in early diagnosis and for evaluation of progressive keratoconus [15].

The ORA have been used to calculate corneal hysteresis (CH) and corneal resistance factor (CRF), which represent two biomechanical corneal parameters. Both parameters are considered reliable to differentiate normal from keratoconic eyes [16]. Recently, the keratoconus match index (KMI) - which is the outcome of seven waveform scores and represents the similarity of the waveform of the examined eye against the same average waveform scores of the keratoconus eyes in the machine’s database-, and the keratoconus match probability (KMP) - which attempts to quantify the probability that a certain eye is actually normal, suspect or keratoconic- estimated by ORA [17]. Labiris et al. estimated that KMI exhibited an overall predictive accuracy of 97.7%, with a sensitivity of 91.18% and specificity of 94.34%. The cut-off point, below which corneas are probably ectatic, was defined as 0.512. Furthermore, they associated the stages 2-3, 3 and 3-4 of Amsler-Krumeich classification with KMP mild, moderate and severe classification, respectively [17].

The OCT pachymetric measurements are accurate and highly repeatable [18, 19]. OCT can overcome limitations of topography resulting from corneal irregularity and tear film break up. Moreover, pachymetric measurements are considered to be more repeatable than those obtained with Scheimpflug imaging in keratoconic eyes [20]. The correlation of OCT-derived epithelial mapping with established Scheimpflug-derived asymmetry topometric indices reveals the OCT reliability in early and advancing keratoconus diagnosis [21].

### 1.3 Treatment of Keratoconus

Although the classic treatment of visual rehabilitation in keratoconus is based on spectacles and contact lenses, the modern surgical techniques include intraocular lenses, collagen cross-linking (CXL), intrastromal implants, microwave remodeling, and anterior lamellar keratoplasty. Collagen cross-linking was clinically introduced in 2003 to delay cone progression, based on photochemical reactions of UVA radiation, riboflavin and oxygen [22]. Until today, it is considered the basic surgical technique to slow keratoconus, changing the steepening of the topographic K-readings in keratoconus patients [23]. The biomechanical modifications achieved with collagen cross-linking are usually accompanied by improvement in visual acuity and spherical equivalent both in younger and individuals over 35 years old [24, 25]. However, haze, bacterial keratitis, corneal melting and band keratopathy are referred as complications of cross-linking and they are estimated to range between 2.6% and 3.9% [23, 25].

Intrastromal corneal ring segments (ICRS) are an alternative treatment for keratoconus in patients with clear corneas and contact lens intolerance [26-28]. The technically demanding manual construction of the intrastromal tunnel for ring implantation was related to complications, including epithelial defects, melting and perforation [26, 27]. The latter explain the attempt to replace the manual technique of tunnel construction by the femtosecond assisted. Ferenczy et al noted that ICRS implanted using femtosecond laser is a safe method to decrease curvature of the cone apex in the topographical analysis, and as well as corrected diopters postoperatively [29].

Penetrating keratoplasty (PK) has been considered as the definitive procedure for the treatment of keratoconus over the past few decades, being accompanied by the risk of allograft endothelial rejection and subsequent risk of graft failure. On the other hand, deep anterior lamellar keratoplasty (DALK) eliminates this risk, involving the removal of anterior diseased cornea and leaving the deeper tissue intact. Femtosecond laser-assisted deep anterior lamellar keratoplasty has been proposed as better surgical option for visual rehabilitation in patients with keratoconus [30]. However, the subsequent final refractive errors and higher-order aberrations may severely affect the visual outcome, even if the surgery is successful [31]. Epikeratoplasty seems to stabilize astigmatism in patients with keratoconus, suffering from intolerance of contact lens, irrespective of astigmatism and corneal thickness [32]. Post-operative complications such as, persistent epithelial defect (PED), severe anterior segment inflammation during early post-operative period, graft melting and interface haze during the intermediate post-operative period, hypoesthesia, peripheral corneal ulcer and posterior sub-capsular cataract during the late post-operative period, have been reported [33-39]. The genes implicated in the pathogenesis of keratoconus open up perspectives for the application of gene therapy in the treatment of the disease [40].

## 2 CONTACT LENSES IN KERATOCONUS

### 2.1 Fitting and Optical Principles of Contact Lenses in Keratoconus

#### 2.1.1 General Fitting Considerations

Keratoconus correction aims to create homogeneous optics in the whole optical system of the eye, free from aberrations of low and high order. Furthermore, the lens choice should be such as, according to the level of ectasia, the keratoconic cornea, to maintain the increased necessities of physiology, keeping in consideration that these patients would ideally use the lenses for long wearing times [41, 42].

Ectatic cornea has decreased tensile properties and reduced thickness as a result of structural alterations at the molecular level of collagen fiber interconnection. It is also a well-known correlation between eye rubbing and acceleration of ectasia. Therefore lenses that are fitted in keratoconic eyes should be in minimum contact with the surface, without applying pressure, because the presence of pressure in conjunction with any rubbing effect could further deteriorate the ectasia or create localized nebulae.

In more advanced cases, the cellular layers, are reduced numerically, adding one more factor of fragility and thus increased need of preserving normal corneal physiology.

Limbus has its importance when contact lens fitting is concerned. The presence of stem cells has an essential role in the corneal physiology and regeneration. Therefore the contact with the limbal area has been minimal, or even absent without rubbing with hard or high modulus hydrophilic materials.

The palpebral conjunctiva is in continuous contact with the lenses in a dynamic manner, between millions of blinks. Therefore contact lens surface has to be well polished, minimizing the interaction between the conjunctiva papillae and the surface or edge of the lens. In the opposite case, the giant papillary conjunctivitis may occur, due to mechanical friction and foreign body material interaction.

### 2.2 Soft Toric or Spherical Lenses *(Fitting Aspects)*

Regardless of mass production or made to order manufacturing procedure, the material is preferred to have high oxygen permeability. The material modulus, important factor in order to assure gentle contact with the corneal and conjunctiva, has to be lower than 0.60MPa. The fit has to be centered with a normal movement of 0.50 mm following blinking.

Spherical or toric lenses, are ideal in cases where optical correction with spectacles, achieves visual acuity of 1.0, free from double images or increased image shadows.

### 2.3 Soft Lenses for Keratoconus or Irregular Cornea *(Fitting Aspects)*

The lenses designed for Ectatic corneas, have increased thickness in optical zone. This varies between 300 to 700 microns. Furthermore, most of the designs incorporate tonic correction and some form of axis stabilization, prismatic or prismodynamic. Therefore in certain areas the lenses are thicker because of the stabilization system [43, 44].

The presence of increased thickness brings the need of materials with high oxygen permeability. Material of Dk more than 80 is preferred. On the other hand, the material modulus has to be the lowest possible, thus below 0.60MPa, because an increased volume lens could become more stressing to the ocular tissues involved.

The combination of the above lens characteristics brings the need of more precise fitting parameters. The base or peripheral curves or the lens diameter are chosen according to the general rules and tests valid for all soft contact lenses [44].

### 2.4 Corneal Rigid Gas Permeable Lenses (RGP) *(Fitting Aspects)*

The corneal RGP lenses are probably the most used globally for the keratoconus cases, for two reasons. They offer a stable optical surface, correcting the optical aberrations through the tear film between the lens and the cornea. Additionally, their manufacturing method is relatively undemanding. They can be produced even with manual lathing technology for the simpler designs. Furthermore, the superb gas permeable materials, offer an excellent option, for more than fifty years, in contact lens technology. They are a reliable alternative even in complex ocular surfaces, when modern CNC technology was not available. An important safety characteristic is that the lens user has to respect the lens or eye physiology limit. Careless handling could lead to irritation which provokes symptoms and the user is obliged to limit the lens wearing schedule.

Historically, the corneal RGP lenses were fitted applying some pressure on the cone area, offering a better high contrast visual acuity and suggesting that mild pressure could contribute to sustain corneal shape. Based on newer physiological data through OCT imaging [45], it is not allowed to fit lenses on keratoconus, applying pressure on the cone, but distributing the eye lens contact to a larger area, permitting tears passing under the lens after each blink. In conjunction with high permeable material of Dk 100 or more, corneal physiology can remain intact.

The palpebral conjunctiva is the tissue that needs extra attention, because depending on the edge design and parameters of lens, could present an increased interaction. Therefore, the optimal edge design is to lift away from the cornea to allow tear passage and at the same time be kept close enough to the surface to avoid mechanical irritation to the mucosal tissue [46, 47].

As far as the limbal area is concerned, in most of the cases, corneal RGP lenses are fitted, limbal area is free from any lens interaction.

### 2.5 Hybrid Lenses with Central RGP and Soft Peripheral Skirt *(Fitting Aspects)*

Hybrid lenses are used in the last decades and have gained preference for their optical correction properties, since they function as RGP lenses but as well as for their initial comfort because of the soft peripheral part [48].

According to their fitting design, the lenses ideally distribute the contact with the tissue evenly, between cornea and conjunctiva or are supported to the peripheral cornea and conjunctiva only. In the latest case the material modulus should be as low as possible in order to respect the corneal and conjunctiva. Because of the soft skirt of the lens, which will dehydrate at a certain level, often a suction effect is present, minimizing any tear circulation that might present during insertion [49].

### 2.6 Scleral Lenses *(Fitting Aspects)*

Scleral lenses are historically the oldest type of lens existing, since middle 1800. At that time, lenses were produced in glass and later in PMMA. The material options were the main reason for their diminished use, blocking any oxygen passage to the cornea [50]. The latest years, scleral lenses have gained a diffuse success for two reasons; the use of high permeable materials of at least 100 Dk and the use of micro precision CNC manufacturing, enabling proper fitting, reproducibility, production of sophisticated designs and customized optical correction, when needed [51, 52].

The fitting principles of scleral lenses are two, but very important to be respected. First the absence of contact with the corneal and limbal region and secondly, the even distribution of contact on the scleral zone that supports the lens. Lens geometries that are in contact with the peripheral cornea or limbus could create symptoms following two to three months wear.

Since the lens almost seals bulbar conjunctiva, minimal tear exchange is occurring under the lens. Therefore, it is important that the user interrupts the wearing schedule every six to nine hours to refill the lens with physiological saline solution [53].

Important aspect of scleral lens design is the lens periphery, anterior and posterior, in order to minimize the lens volume, maintaining the stability and reducing the lens lid interaction [54, 55].

Optical correction principles and lens characteristics.

### 2.7 Soft Lenses for Keratoconus *(Optical Correction)*

Hydro gel lens for the Ectatic corneas are widely adopted for the leading practitioners but as well as for the patients, for their comfort. Further advantages consist of prolonged wearing schedule and possibility of fitting them soon after CXL treatment, interfering minimally to the strengthening therapy of the cornea. In addition, they can be used in conjunction with RGP lenses for more dynamic or leisure activities, when comfort is more important.

Their correcting principle is based on creating an external optical surface (the anterior of the lens) more homogeneous, topographically and thus with less high order aberrations. The topographical blending is achieved through the increased thickness of the optical zone and the front surface asphericity. In addition with the correction of low order aberrations (spherical and cylinder correction), the keratoconic eye can achieve an improved or optimal visual acuity.

In more sophisticated lens types, asymmetric optical corrections could be incorporated, correcting the main optical aberration in keratoconus, the vertical coma. On these lenses optical power is different on the superior and inferior part of the optical zone. For example, the upper part could have a power of -1.00 D and the lower -5.00 D [56].

Controlling objectively the result of these lenses is straightforward, performing a corneal topography over the lens in vivo. Any residual surface aberration is evident, which in some cases could contribute to neutralize the ones created from the posterior corneal surface.

Technical characteristics include increased optical zone thickness from 300 to 700 microns, spherical correction from -40.00 to +30.00 D, cylindrical correction up to 12.00 DC, base curve more commonly from 8.00 to 9.20 mm. The materials used are hydrogels with medium to high water content, or silicone hydrogels with Dk of 50 to 60.

### 2.8 Corneal Rigid Gas Permeable Lenses *(Optical Correction)*

The corneal RGP lenses are the most widely used lenses for the keratoconus cases, providing optimal high contrast visual acuity. Moreover they are easily fitted by experienced practitioners due to the fact that fitting is evident with fluorescein staining the tear film under the lens. Over refraction is straightforward using spherical correction to the majority of the cases. From the optical point of view, low order astigmatism and high order aberrations are corrected with the tear fluid under the lens. In many cases, a gentle touch of the cone area, corrects the residual coma of the posterior corneal surface [57, 58].

Disadvantage of the corneal RGP lenses is the quality of vision when the lens is not centered properly or tilt in the lens is present. Resultant high order aberrations might be present and even if Snellen acuity is optimal, contrast sensitivity is reduced.

To improve the result, current contact lens designs have large optical zones (7.50 to 8.00 mm). In addition, they use aspheric surfaces to coincident to the ectatic cornea, with increased lens diameters up to 10.00 or 11.00 mm. The fitting of large diameter lenses is becoming more complicated though, because, it could lead to the areas of increased unwanted pressure on the cornea [59-60].

### 2.9 Hybrid Lenses *(Optical Correction)*

Hybrid lenses usually are composed of a rigid part of 8.00 mm in the centre and a flexible hydrogel part with 14.50 mm of total diameter. The correction principle is identical to the corneal RGP lenses described above. Some designs claim to imitate the correction principle of scleral lenses, having a distance from the central cornea [61]. Hybrid lenses are well centered due to their soft skirt providing a centered optical correction. The over refraction in these lenses is spherical, so there is no possibility of incorporating more complex toric or asymmetric aberrations correction.

### 2.10 Scleral Lenses *(Optical Correction)*

In the latest years, more sophisticated designs of lenses enable the practitioners to fit relatively smaller lens diameters [61], in respect to the past, of 16 to 20 mm, yet achieving optimal fit for the most demanding cases. In general, the diameter of scleral lens varies between 16 to 24 mm . Generally accepted minimal diameter in order to have a corneal and limbal area free from pressure is 16 mm. The optical correction with this type of lenses is achieved with the liquid, well-centered space, between lens and cornea. Therefore any optical aberration of the front corneal surface is neutralized. Disadvantage of the scleral lens correction is the remaining aberrations from the back corneal surface. Modern lens designs incorporate two options; front custom aspheric surface, correcting spherical aberration or front toric correction, manufactured on the front surface of the lens. The stable and highly gas permeable material assured the optical result throughout the wearing schedule, maintaining at the same time continuous corneal lubrication and protection [50, 51].

## CONCLUSION

The correction of refractive disorders in keratoconus, using lenses, includes gas permeable (GP) contact lenses, soft or scleral lenses, hybrid contact lenses and piggyback systems. GP lenses range in size from 8.0 to 10.0 mm in diameter, fitting better in small central or mild cones [62]. They have been found to achieve better best corrected visual acuity (BCVA) compared to spectacles, depending on their types (51.85%, 43.39%, 4.23% and 0.53% in rigid gas permeable (RGP), semi-gas permeable (SGP), polymethyl methacrylate (PMMA) and hard-soft gas permeable contact lenses, correspondingly). Furthermore, they have been estimated to eliminate 3rd-order coma improving the quality of vision. Both an apical-touch and three-point-touch fitting seem to reduce corneal asphericity and astigmatism and lower higher-order aberrations. However, they have implicated in the reduction of corneal basal epithelial cell and anterior stromal keratocyte densities [54, 63].

Various soft contact lenses, with base curve between 6.0 to 7.0 mm, are indicated in early or decentered keratoconus and in GP intolerance. Although they provide greater comfort, low oxygen permeability, (with the exception of silicone hydrogel), and the inability to mask moderate to severe irregular astigmatism are major disadvantages of soft contact lenses. On the other hand, scleral contact lenses ensure stable platforms, which eliminate high-order aberrations and provide good centration and visual acuity. However, prolonged discomfort period and handling complexity limit their use. Furthermore, they induce corneal changes, including corneal flattening scarring and weakening [64, 65].

Modern hybrid contact lenses, are usually chosen when there is poor centration, poor stability or intolerance with GP lenses. Nevertheless, they require special training to fit. Finally, piggyback contact lens systems are an alternative for patients who exhibit intolerance to scleral or rigid corneal lenses. Although silicon hydrogel contact lenses are widely used in these systems, Dk RGP lens materials and aspheric designs are available, providing the cornea, a required amount of oxygen. Furthermore, they improve vision, correcting astigmatism and the high-order aberrations. In piggyback users, corneal epithelium and endothelium seem; to be unaffected. However neovascularization and giant papillary conjunctivitis have been reported in few patients [61, 62, 66, 67].
